# Preferred Learning Styles and Teaching Methods Among Medical Students: A Cross-Sectional Study

**DOI:** 10.7759/cureus.46875

**Published:** 2023-10-11

**Authors:** Waad Alfarsi, Arwa H Elaghoury, Smitha Elizabeth Kore

**Affiliations:** 1 Medicine, College of Medicine and Health Sciences, National University of Science and Technology, Sohar, OMN; 2 Anatomy & Neurobiology, College of Medicine and Health Sciences, National University of Science and Technology, Sohar, OMN

**Keywords:** medical students, preference, teaching methods, vark questionnaire, learning style

## Abstract

Introduction

Knowing the learning styles of medical students is an important factor as it can help in making learning more effective. Research has shown that medical students in various years of studies have selective learning styles and liking to a particular teaching strategy. The aim of the present study was to identify the preferred learning styles and teaching methods of medical students through all the years of study using the VARK (for Visual, Aural, Read/Write, Kinesthetic) questionnaire.

Method

A cross-sectional study was conducted among the medical students of year 1 (MD1) to year 6 (MD6), except MD5, at the College of Medicine and Health Sciences, Sohar, Oman. The VARK questionnaire was used to record the learning style preference of the students, and an additional question was asked to know the teaching method that the students preferred to be used.

Results

A total of 292 students responded to the questionnaire. The majority of the students had preference for the multimodal style of teaching (54%), mostly bimodal (30%). This was followed by the kinesthetic mode of learning (30%) in the unimodal style. The MD1 and MD6 students had preference for the unimodal style whereas the other years (MD2, MD3, MD4) had preference for the bimodal style. The teaching method preferred by most of the students included clinical skills lab (64%) followed by interactive lecture (59%) and lab work (57%).

Conclusion

The study showed that most of the students had preference for more than one (bimodal) learning style. The research findings can help the teachers recognize the learning problems among students and execute the needed teaching strategies.

## Introduction

Learning styles are habits adapted by the learners to perceive, analyze, and interpret their knowledge [1-3]. Each student has their own learning style, which is usually determined by their type of personality or how they conceive learning and the environment they are placed in [4,5]. Medical students during their course of study are exposed to various teaching strategies that include lectures, lab work, small group discussions, case-based learning, bedside teaching, etc. Their study method changes as they progress from the preclinical to clinical years of study. Knowledge of the students learning style can aid a teacher in making learning more effective, as no one teaching strategy can cater to the needs of all students [6]. Furthermore, determining learning styles among students can also help the teacher strengthen the students' preferred learning style and help in improving other non-preferred learning styles. Also, if the curriculum design is adapted to the students’ learning preferences and styles, their motivation and performance could improve [1].

Researchers have classified the learning styles based on different approaches. Some are based on the bipolar construct, and others on the depth of learning [7,8]. In the present study, a sensory model, the VARK inventory (developed by Fleming and Mills; VARK Learn Limited, New Zealand), which stands for Visual, Auditory, Read/Write and Kinesthetic modalities, was used [3,9]. This model is widely accepted by students, teachers, and researchers [10]. This is attributed to its simplicity, ease of access and excellent reliability and validity [11].

VARK allows for a better understanding of information-processing preferences, including a learner's ability to use more than one learning mode simultaneously [1]. It is composed of 16 questions that focus on content delivery and communication with others. Visual learners are able to handle information better when they can see it. They prefer the use of charts, diagrams, mind maps, videos, etc. Those who are auditory learners prefer to hear the information. For example, they benefit the most from lectures, recordings, and discussions. Read/write students favor the teaching material displayed in words. They are more interested in designing and reviewing notes as well as reading textbooks several times. The kinesthetic learners learn best with practice and simulation or any experience that connects the material to reality. A multimodal pattern incorporates the students who can acquire or process information through more than one learning style [3].

In addition to addressing students' learning style, the study focuses on determining the preferred teaching methods as these two are closely related to each other [1]. The quality of teaching environment and assessment procedures influence students' methods of learning and the outcomes [4,5,8,9]. Surface approaches, as in using one mode of studying based on the VARK survey, were manifested in an educational environment that puts the learners under overwhelming class demands [8]. In King Saud University, medical students who were unimodal learners favored the aural (auditory) mode and they linked this to the fact that Saudi Arabian high schools are teacher centered and lecture based [1].

The traditional lecture-based teaching method is one of the oldest methods of medical teaching; these methods are teacher centered but are gradually phasing out with many institutions now mostly preferring active teaching methods like problem-based learning that enables the students to be self-directed learners. No single instructional strategy can be used for all types of learning styles and is not suitable for the learning process [7].

In 2014, a study on preclinical medical students in the present institution in Oman showed that the majority of the students preferred a multimodal approach [12]. The study was limited to the preclinical years, unlike the present study that included the study of preferred learning styles of both the preclinical and clinical years.

The aims of the present study were to identify the preferred learning styles of medical students through all the years (MD1-MD6) of study through the VARK questionnaire and to determine the preferred teaching methods in each year of study.

## Materials and methods

A cross-sectional study was conducted to know the preferred learning styles and teaching methods among the year 1 (MD1) to year 6 (MD6), except MD5, students at the College of Medicine and Health Sciences, Sohar. Convenience sampling was used and all the students who were willing to participate in the study were included after taking informed consent. Ethical approval for the study was obtained from the Ethics & Biosafety Committee, College of Medicine and Health Sciences (NU/COMHS/EBC003/2022).

To study the learning styles of students, a pre-validated VARK questionnaire (version 8.01) was used [13]. The questionnaire consists of 16 questions with a choice of four options that can be chosen, with each of the options corresponding to a sensory modality. Students had the chance to select one or more than one option; thus, a varied combination of sensory modalities was obtained. The questionnaire was released online as a Google Form via email where the link to fill the questionnaire online was shared; the students after filling the questionnaire entered their score in the space provided in the Google Form.

An additional question was asked in the Google Form (Google, Mountain View, CA) to know the teaching methodology that was preferred by the students. The options for the question consisted of all the teaching methodologies used in the institute in different years of study. The student had the freedom to select more than one option.

The data collected was statistically analyzed using the IBM SPSS software, version 25 (IBM Corp., Armonk, NY). Descriptive statistics were employed to know the frequencies of the responses. Chi-square values were was also tested to explore associations among variables in the different years of study.

## Results

The total number of students who responded to the questionnaire was 292. The majority of responses were obtained from year 2 (MD2) students (87 responses). The number of students who participated in the study from each year is shown in Table 1, along with all the teaching methodologies used in the institute. Responses from MD5 students were excluded from the study due to insignificant numbers.

**Table 1:** Preferred teaching methods from each year of study The number of responses from each year is mentioned in parentheses.

S. no.	Teaching method	MD1 (32)	MD2 (87)	MD3 (57)	MD4 (75)	MD6 (41)
1	Interactive lecture	63%	55%	54%	52%	80%
2	Small group discussion (SGD)	53%	44%	33%	28%	46%
3	Demonstration on models	38%	61%	60%	61%	41%
4	Student presentation	3%	9%	5%	7%	7%
5	Lab work	91%	68%	40%	57%	32%
6	Problem-based learning (PBL)	41%	26%	30%	25%	24%
7	Case-based learning (CBL)	25%	37%	26%	13%	22%
8	Team-based learning (TBL)	22%	30%	32%	27%	37%
9	Guest speaker	22%	13%	18%	15%	10%
10	Clinical skills lab	63%	72%	54%	71%	49%
11	Bedside teaching	47%	44%	42%	73%	76%
12	Intradepartmental lecture (IDL)	6%	8%	19%	15%	22%
13	Online teaching	25%	47%	37%	25%	7%
14	Self-directed learning (SDL)	44%	49%	23%	33%	20

**Figure 1:**
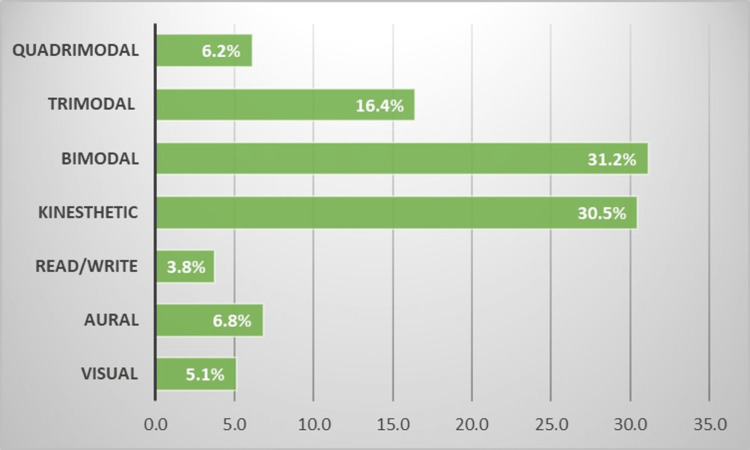
Preferred learning styles of students (no. of responses = 292)

**Figure 2:**
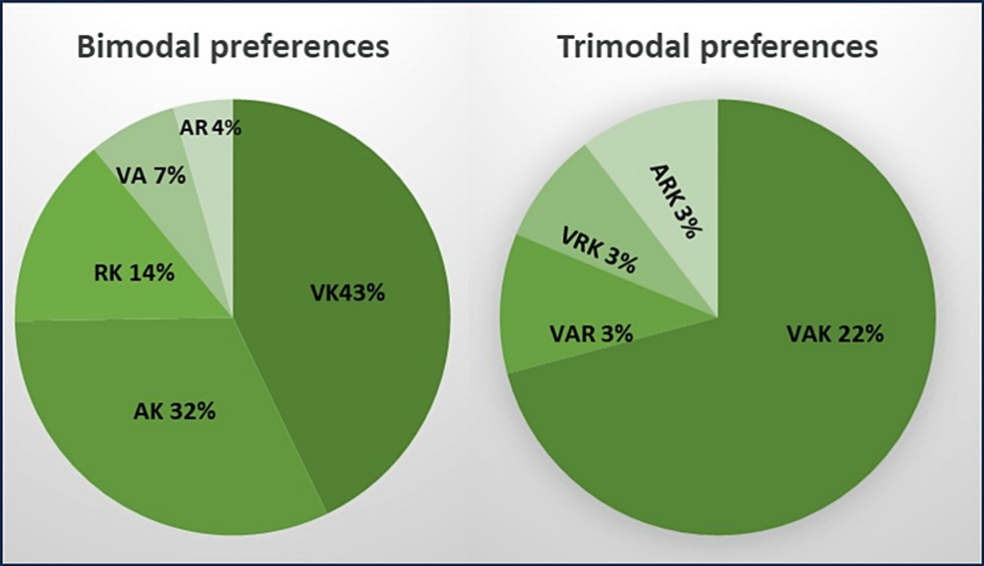
Preferred learning styles of students with bimodal and trimodal approaches V, visual; A, aural; R, read/write; K, kinesthetic

Out of the 292 students who responded to the questionnaire, the majority of the students had preference for a multimodal approach (54%) out of which the bimodal approach was the most preferred one (31%) (Figure 1). The visual and kinesthetic (43%) approach was the most preferred in the bimodal approach followed by the aural and kinesthetic approach (32%) as shown in Figure 2.

In the unimodal approach (46%), one-third (30%) of students showed a preference for the kinesthetic approach (Figure 1).

**Figure 3:**
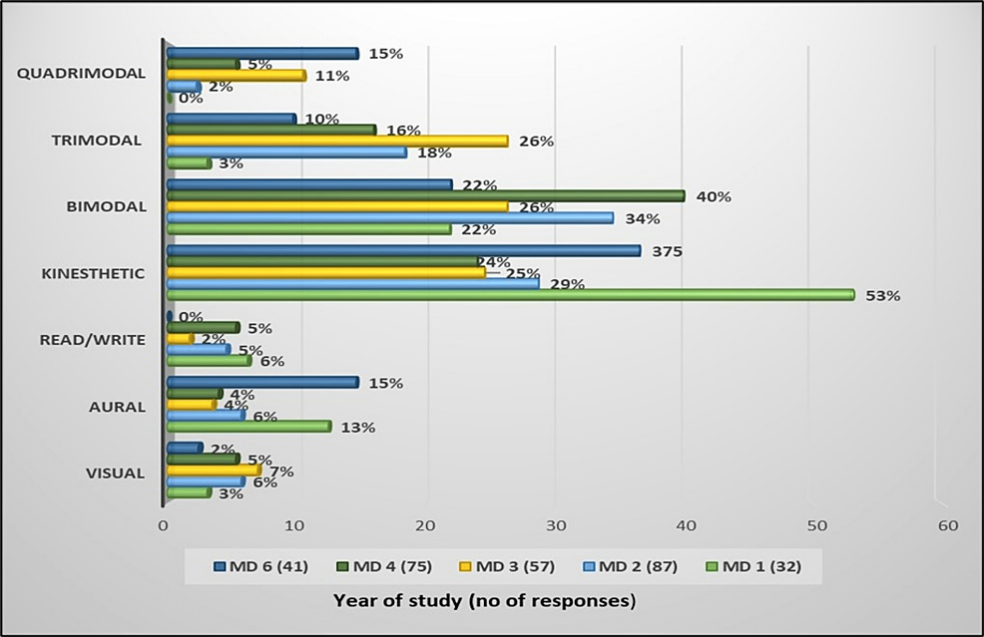
Preferred learning styles of students from each year of study

In the various years of study, the students from MD1 and MD6 had a higher preference for the unimodal approach of learning, with the kinesthetic approach being the most preferred, whereas the students of MD2, MD3 and MD4 had a preference for the multimodal approach (Figure 3).

**Figure 4:**
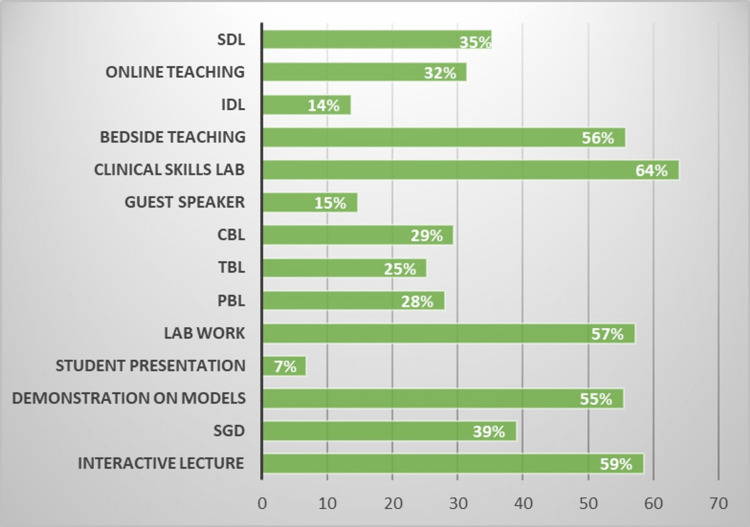
Preferred teaching methods of students (no. of responses = 292) SDL, self-directed learning; IDL, intradepartmental lecture; CBL, case-based learning; TBL, team-based learning; PBL, problem-based learning; SGD, small group discussion

The teaching style that was most preferred by the 292 students was the clinical skills lab (64%) followed by interactive lecture (59%) and lab work (57%) (Figure 4).

MD1 and MD6 students who had preferred the kinesthetic approach the most preferred teaching strategies that included lab work (91%, MD1) and interactive lecture (80%, MD6) followed by bedside teaching (76%) whereas the preferred teaching method of MD2 students was clinical skills lab (72%) followed by lab work (68%); for MD3 students, it was demonstration on models (60%), followed by clinical skills lab and interactive lecture (54%). MD4 students preferred bedside teaching (73%) followed by clinical skills lab (71%), as shown in Table 1.

Chi-square analysis showed no significant inter-item association between the results for various years of study.

## Discussion

The current cross-sectional study aimed at determining the preferred learning style and teaching methods among medical students. Findings illustrated a preponderance of multimodal style in MD2, MD3 and MD4 students. Similar observations were noted by studies conducted in the United States by El Tantawi and Murphy et al. [14,15]. Urval et al. assessed the learning style and its influence on the sex and academic outcomes in medical students; their study revealed that the most common preference was multimodal [16]. A study conducted in Saudi Arabia reported that multimodal style was confined to level 1 students because the students came from preparatory schools where learning using a variety of modalities was dominant [17]. Another study reported that the students who were context-specific learners tended to be multimodal, switching between learning modes depending on the subject. In addition, multimodal learners could be the ones who are not satisfied with their learning experience until they apply all their preferred learning styles [12].

The unimodal approach was the preferred modality of learning in MD1 and MD6 students, accounting for 75% and 54%, respectively. The results of the present research are in part similar to those of Bokhari and Zafar and Wong et al. [6,18]. However, in the former study, they found that as the students advanced in their academic years, they shifted toward the multimodal approach. They attributed this switch to the nature of the medical curriculum in advanced years that necessitated the students to utilize different styles of learning due to the introduction of problem-based learning, discussion and practical skills and reduction in the number of didactic lectures [7]. Studies conducted by El Sayed et al. and Naggar reported that as the students progress in their medical school, they tend to switch to the unimodal learning style [19,20]. These variations among different medical schools may be due to the material studied, teaching methods and assessment strategies.

Among the learning modalities V, A, R, and K described in this study, the most preferred modality was kinesthetic [21]. This learning style was second to the aural approach in the University of Bisha in Saudi Arabia; the kinesthetic learning style is either the most dominant or the second most dominant style in studies conducted in Saudi Arabia [17]. However, a similar type of study conducted in 2014 that included preclinical medical students at the institute of the present study illustrated that the unimodal learners were homogeneously distributed across the four categories [12]. Since this study, the curriculum of the medical college has undergone a variety of modifications and this might be the reason for the change in students' preference. Pursuant to Cebeci et al., learning style can be influenced by the quality of the teaching environment and assessment system [8].

Teaching methods greatly influence the learning process and modern teachers need to use more innovative educational technologies to cater to the needs of the tech-savvy students of the present generation. Patra et al. described a variety of advanced teaching methods like anatomy studio, web-based training, 3D stereoscopy, virtual dissection, etc., to facilitate learning of anatomy, and that can definitely be considered while teaching other courses in medical studies [22].

In our study, participants were also asked to indicate their preferences in terms of the teaching methodology. The preferred teaching methods varied between the years, but there was a preponderance of methods that incorporated active learning such as clinical skills lab (64%), interactive lectures (58%), lab work (57%), bedside teaching (56%) and demonstration on models (55%). These results were compatible with the once observed by Bhalli et al. and Costa et al. who found that the majority of the students preferred interactive lectures [7,23]. The least favored styles were those that involved didactic or one-way teaching such as students' presentations, intradepartmental lecture (IDL) or guest lecture. Same results were observed by Bhalli et al. and Mukhtar et al. [7,24].

Bedside teaching and clinical skills labs accounted for a large number of responses from MD4 students achieving a percentage of 73% and 71%, respectively. These teaching strategies were only introduced to the clinical years (MD5 and MD6), but they were noted to attain quite a number of responses from the preclinical years (MD1, MD2, MD3 and MD4). The authors believe that the students not only selected the preferred teaching method they experienced during their academic years but also selected the methods that they wished to be added to their current course in the future.

## Conclusions

This study revealed that students in their first and last year of study preferred mostly the unimodal kinesthetic type of learning style whereas the multimodal approach, mainly bimodal (visual and kinesthetic), was preferred by the students in other years of study. Most of the students also opted for interactive teaching strategies and skill-based clinical learning that went along well with their preference of learning style. It would benefit the students if the teachers went along with the preferences of the students and made the necessary changes in their teaching strategies to facilitate learning. In addition, using multiple teaching strategies can also cater to the needs of students with various learning styles. The institute may also regularly administer the present questionnaire so that student-favored learning styles may be understood and the needed teaching strategy be introduced.

## References

[1] Almigbal TH (2015). Relationship between the learning style preferences of medical students and academic achievement. Saudi Med J.

[2] Lujan HL, DiCarlo SE (2006). First-year medical students prefer multiple learning styles. Adv Physiol Educ.

[3] Liew SC, Sidhu J, Barua A (2015). The relationship between learning preferences (styles and approaches) and learning outcomes among pre-clinical undergraduate medical students. BMC Med Educ.

[4] Bonsaksen T (2018). Factors associated with occupational therapy students’ preferences for courses and teaching. Cogent Educ.

[5] Bin Abdulrahman KA, Khalaf AM, Bin Abbas FB, Alanazi OT (2021). Study habits of highly effective medical students. Adv Med Educ Pract.

[6] Bokhari NM, Zafar M (2019). Learning styles and approaches among medical education participants. J Educ Health Promot.

[7] Bhalli MA, Khan IA, Sattar A (2015). Learning style of medical students and its correlation with preferred teaching methodologies and academic achievement. J Ayub Med Coll Abbottabad.

[8] Cebeci S, Dane S, Kaya M, Yigitoglu R (2013). Medical students’ approaches to learning and study skills. Procedia Soc Behav Sci.

[9] Mozaffari HR, Janatolmakan M, Sharifi R, Ghandinejad F, Andayeshgar B, Khatony A (2020). The relationship between the VARK learning styles and academic achievement in dental students. Adv Med Educ Pract.

[10] Hernandez JE, Vasan N, Huff S, Melovitz-Vasan C (2020). Learning styles/preferences among medical students: kinesthetic learner's multimodal approach to learning anatomy. Med Sci Educ.

[11] Bin Eid A, Almizani M, Alzahrani A (2021). Examining learning styles with gender comparison among medical students of a Saudi University. Adv Med Educ Pract.

[12] Panambur S, Nambiar V, Heming T (2014). Learning style preferences of preclinical medical students in Oman. Oman Med J.

[13] (2023). VARK questionnaire: how do you learn best?. https://vark-learn.com/the-vark-questionnaire/.

[14] El Tantawi MM (2009). Factors affecting postgraduate dental students’ performance in a biostatistics and research design course. J Dent Educ.

[15] Murphy RJ, Gray SA, Straja SR, Bogert MC (2004). Student learning preferences and teaching implications. J Dent Educ.

[16] Urval RP, Kamath A, Ullal S, Shenoy AK, Shenoy N, Udupa LA (2014). Assessment of learning styles of undergraduate medical students using the VARK questionnaire and the influence of sex and academic performance. AJP Adv Physiol Educ.

[17] Rezigalla AA, Ahmed OY (2019). Learning style preferences among medical students in the College of Medicine, University of Bisha, Saudi Arabia (2018). Adv Med Educ Pract.

[18] Wong RSY, Siow HL, Kumarasamy V, Shaherah Fadhlullah Suhaimi N (2017). Interdisciplinary and inter-institutional differences in learning preferences among Malaysian medical and health sciences students. J Adv Med Educ Prof.

[19] El Sayed M, Mohsen D, Dogheim R, Zain H, Ahmed D (2016). Assessment of learning styles for medical students using VARK questionnaire. Int J Manage Appl Sci.

[20] Naggar ME (2016). Identifying and comparing learning styles preferences among medical undergraduates students at College of Medicine Aljouf University. Intel Prop Rights.

[21] Asad MR, Asghar A, Tadvi N (2023). Medical faculty perspectives toward cadaveric dissection as a learning tool for anatomy education: a survey study in India. Cureus.

[22] Patra A, Asghar A, Chaudhary P, Ravi KS (2022). Integration of innovative educational technologies in anatomy teaching: new normal in anatomy education. Surg Radiol Anat.

[23] Costa ML, van Rensburg L, Rushton N (2007). Does teaching style matter? A randomised trial of group discussion versus lectures in orthopaedic undergraduate teaching. Med Educ.

[24] Mukhtar F, Hashmi N, Rauf MA, Anzar A, Butt KI, Ahmed M, Abbas K (2012). Teaching methodologies; what is the students’ perspective?. Prof Med J.

